# Solute carrier 41A3 encodes for a mitochondrial Mg^2+^ efflux system

**DOI:** 10.1038/srep27999

**Published:** 2016-06-15

**Authors:** Lucia Mastrototaro, Alina Smorodchenko, Jörg R. Aschenbach, Martin Kolisek, Gerhard Sponder

**Affiliations:** 1 Institute of Veterinary-Physiology, Free University of Berlin, Oertzenweg 19b, Berlin, D-14163, Germany; 2 Institute of Vegetative Anatomy, Charité, Universitätsmedizin Berlin, Campus Charité-Mitte, Berlin, D-10117, Germany

## Abstract

The important role of magnesium (Mg^2+^) in normal cellular physiology requires flexible, yet tightly regulated, intracellular Mg^2+^ homeostasis (IMH). However, only little is known about Mg^2+^ transporters of subcellular compartments such as mitochondria, despite their obvious importance for the deposition and reposition of intracellular Mg^2+^ pools. In particular, knowledge about mechanisms responsible for extrusion of Mg^2+^ from mitochondria is lacking. Based on circumstantial evidence, two possible mechanisms of Mg^2+^ release from mitochondria were predicted: (1) Mg^2+^ efflux coupled to ATP translocation via the ATP-Mg/Pi carrier, and (2) Mg^2+^ efflux via a H^+^/Mg^2+^ exchanger. Regardless, the identity of the H^+^-coupled Mg^2+^ efflux system is unknown. We demonstrate here that member A3 of solute carrier (SLC) family 41 is a mitochondrial Mg^2+^ efflux system. Mitochondria of HEK293 cells overexpressing SLC41A3 exhibit a 60% increase in the extrusion of Mg^2+^ compared with control cells. This efflux mechanism is Na^+^-dependent and temperature sensitive. Our data identify SLC41A3 as the first mammalian mitochondrial Mg^2+^ efflux system, which greatly enhances our understanding of intracellular Mg^2+^ homeostasis.

The key role of magnesium (Mg^2+^) in a plethora of biochemical processes requires the tight regulation of intracellular Mg^2+^ homeostasis (IMH). The intracellular Mg^2+^ concentration [Mg^2+^]_i_ is regulated by the modulation of cellular uptake and efflux and by intracellular storage^1^. Several channels or transporters have been characterized as mediating the uptake of Mg^2+^ (e.g., TRPM6/7, MagT1, or NIPA1) or its extrusion (SLC41A1) across the cytoplasmic membrane^2^,^3^,^4^,^5^.

Member A1 of the solute carrier family 41 (SLC41A1; further referred to as A1) has been characterized as being a ubiquitous Na^+^-dependent Mg^2+^ efflux system integral to the plasma membrane^6^,^5^. Whereas the characterization of plasma membrane-localised Mg^2+^ transporters is improving, the transporters for intracellular Mg^2+^ are largely unexplored. To date, the mitochondrial channel Mrs2^7^,^8^ and Golgi-localised MMgt1/2^9^ are the only known Mg^2+^ transport systems integral to membranes of subcellular compartments. Their discovery supports an earlier assumption that mitochondria, the endoplasmic reticulum (ER), and the Golgi apparatus serve as intracellular Mg^2+^ stores^10^,^11^.

Kubota and colleagues have documented the release of Mg^2+^ from mitochondria upon their depolarization in PC12 cells^10^. Furthermore, long-chain fatty acids induce the rapid release of Mg^2+^ from rat liver mitochondria in alkaline media, presumably via an Mg^2+^/Me^+^ or an Mg^2+^/H^+^ exchanger^12^. Despite the increasing evidence in favour of such a mitochondrial exchanger (Mg^2+^ efflux system), its molecular identity in mammalian cells is as yet unknown. Recently, Cui *et al.* have characterized the protein Ymr166c/Mme1 (mitochondrial magnesium exporter 1) as the first known Mg^2+^ exporter in yeast mitochondria. Reconstitution experiments in proteoliposomes have shown that this transport activity is dependent on the presence of ATP, although ATP hydrolysis is not required. The authors therefore speculate that Mme1 acts as ATP/ATP-Mg exchanger^13^. In a subsequent study, the same group has identified the gene *CG3476* as a *Drosophila melanogaster* orthologue of Mme1. The heterologous expression of *CG3476* in yeast significantly reduces their mitochondrial Mg^2+^ levels. Knock-down or overexpression of the gene both reduces the viability of *Drosophila*. However, the precise mode of function of Ymr166c/Mme1 remains to be elucidated^14^.

The present study was based on the hypothesis that SLC41A3 can carry out Mg^2+^ export in eukaryotic cells, similarly to that of SLC41A1. Like the two other members of solute carrier family 41 (SLC41A1 and A2) SLC41A3 contains two “MgtE-like domains” displaying homology to the prokaryotic Mg^2+^ transporter MgtE^15^,^16^. Human *SLC41A3* has been mapped to chromosome 3q21.2–q21.3, and seven alternative splice variants are predicted to be produced. The full-length protein consists of 507 amino acids with a molecular weight of 54.7 kDa. *SLC41A3* transcripts have been detected in various murine tissues, with the highest levels occurring in the central nervous system (in particular, in the neuronal cells of the cerebral cortex, the hippocampus, and lateral ventricle and in most cell types of the cerebellum)^17^. The anticipated importance of SLC41A3 (further referred to as A3) in CNS is further supported by the detection of neurological and behavioural abnormalities and, in particular, the abnormal locomotor coordination with ataxia, in the conditional knock-out mouse line *Slc41a3*^*tm1a(KOMP)Wtsi*^ (http://www.mousephenotype.org/). Furthermore, the expression of *A3* is significantly increased in mice fed a Mg^2+^-deficient diet suggesting the importance of A3 for IMH^18^,^19^. The heterologous overexpression of A3 in *Xenopus* oocytes is associated with electrogenic Mg^2+^ conductance. These currents are saturable with a *K*_m_ of 1.5 mM. Moreover, other ions have been identified as being transported via A3, a finding suggesting that A3 is an unspecific cation channel with a broad permeation profile^20^. The A3-specific electrogenic Mg^2+^ conductance seen in *Xenopus* oocytes has not been detected in mammalian cells. Sahni and colleagues have reported that A3 overexpressed in TRPM7-deficient B lymphocytes fails to restore normal Mg^2+^ homeostasis in these cells^15^.

We have therefore examined the cellular localisation of A3 and its ability to transport Mg^2+^. The presented data provide convincing evidence that SLC41A3 encodes a mitochondrial Mg^2+^ transporter responsible for the Na^+^-dependent efflux of the ion.

## Results

### Creation of tet-inducible HEK293 cell lines overexpressing SLC41A3

**Figure 1:**
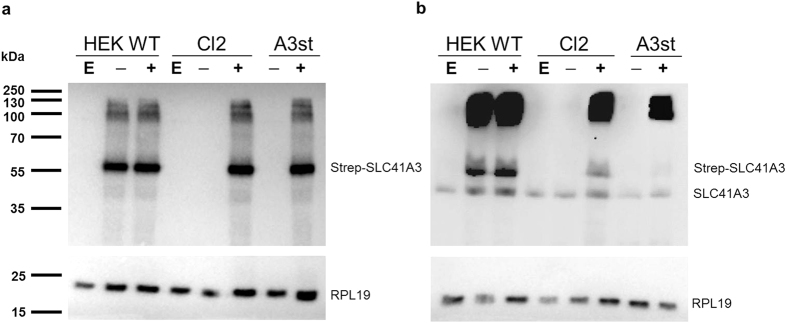
Comparison of SLC41A3 protein levels in various HEK293 cell lines. HEK293 WT cells (HEK WT) or the HEK293 cell line with stably integrated Tet-repressor (Cl2) were transiently transfected with pcDNA5TO-SLC41A3 (− and +) or the empty vector control (E). Protein expression in the transiently transfected cell lines (HEK WT and Cl2) and in the stably transfected cell line (A3st) was induced by addition of tetracycline to a final concentration of 1 μg/ml (+). Uninduced control cells remained untreated (−). Cells were harvested after 24 hours of induction and lysed in RIPA buffer. Total protein extracts were analyzed on an 8.5% PAA-SDS gel and immunodetection of SLC41A3 (55 kDa) was either performed with an antibody directed against the Strep-tag (**a**) or against the native protein (**b**). The signals for the monomeric forms of native and Strep-tagged SLC41A3 are indicated. The ribosomal protein RPL19 (23 kDa) served as loading control and was detected by reprobing the membranes with the respective antibody.

To study the function and cellular localisation of A3, a tet-inducible stably transfected cell line was newly generated expressing the protein with an N-terminal HA-Strep tag (A3st). At 24 hours after the addition of tetracycline, efficient inducible expression was detected by Western blot analysis with an antibody against the Strep tag (Fig. 1a, A3st). Furthermore, a HEK293 cell line stably expressing the tetracycline repressor (tetR) was constructed that served as a host for the regulated transient expression of A3. Wild-type HEK293 cells were transfected with the linearized plasmid pcDNA6/TR and cultured under blasticidin selection. Clones that stably expressed the tet repressor were subsequently transfected with the pcDNA5/TO-SLC41A3 construct and tested for their ability to down-regulate the expression of A3 in the absence of tetracycline. Amongst all transiently A3-expressing cell lines, “Clone2” (Cl2) exhibited the lowest level of A3 expression in the absence of tetracycline and abundant expression after induction and was therefore used for further experimentation (Fig. 1a). In contrast to Cl2, protein expression in HEK293 wild-type cells transiently transfected with pcDNA5/TO-SLC41A3 (HEK WT) was independent of the presence of tetracycline due to the absence of the tet repressor (Fig. 1a). We furthermore sought to compare the expression levels of native and overexpressed A3. In parallel to the detection of overexpressed A3, the same samples were analysed on a parallel blot with an antibody directed against native A3 (Fig. 1b). Specificity of this antibody was confirmed in a blocking peptide competition assay (Supplemental Fig. S1). Immunodetection with the A3 antibody yielded two different signals, one for the monomeric form of untagged and one for the monomeric form of Strep-tagged A3. The apparent size difference is due to the HA-Strep tag which increases the molecular weight of SLC41A3 by approx. 4,3 kDa (Fig. 1b). Both antibodies furthermore detected high molecular weight signals between 100 and 250 kDa (Fig. 1a,b). These signals might arise from A3-containing, protein complexes that are not readily dissolved in sample loading buffer. Alternatively, these signals might be A3 aggregates resulting from the strong overexpression of the protein.

### SLC41A3 does not mediate Mg^2+^ transport across the plasma membrane but leads to an increase in the free cytoplasmic [Mg^2+^] when overexpressed

**Figure 2:**
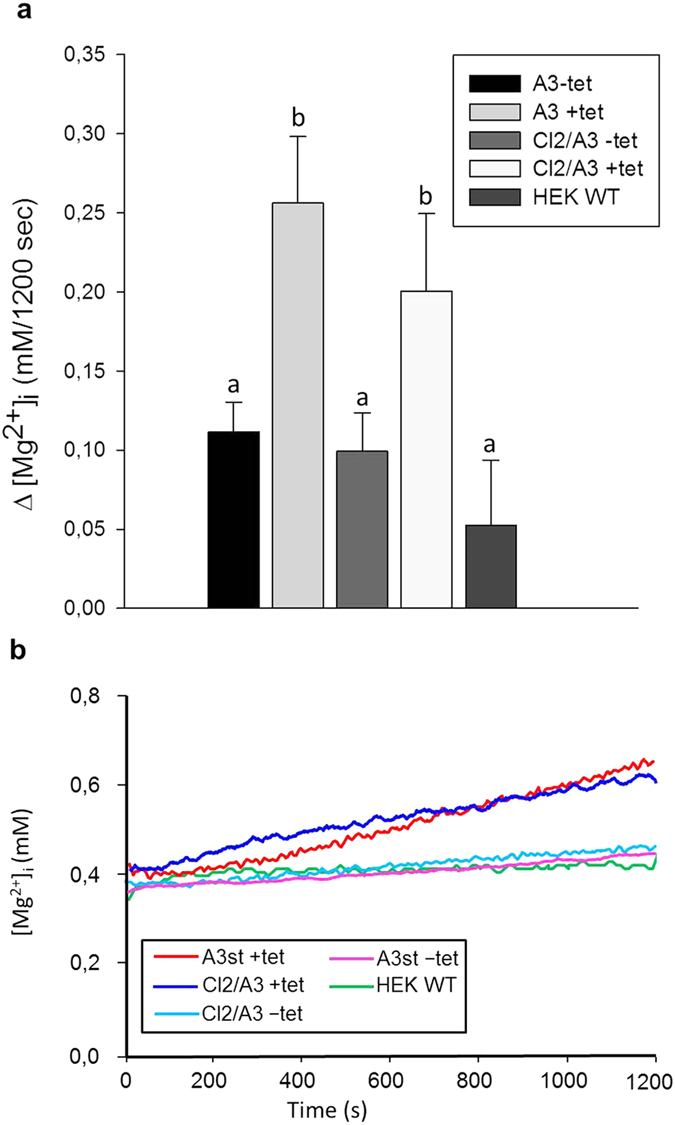
Effect of the expression of SLC41A3 on the change of the intracellular free magnesium concentration (Δ[Mg^2+^]_i_) in HEK293 cells. **(a)** Tetracycline-induced (+tet) and uninduced (−tet) cells of the SLC41A3 overexpressing cell lines A3st (stable A3 expression) and Clone2 (transient A3 expression) were loaded with Mg^2+^, and changes of [Mg^2+^]_i_ after 1,200 s in completely Mg^2+^-free solution were determined. Wild-type HEK293 cells (HEK WT) served as a control. Values are given as means ± SEM. Number of measurements: N_A3 −tet_ = 17; N_A3 +tet_ = 12; N_Cl2/A3 −tet_ = 10; N_Cl2/A3 +tet_ = 10; N_HEK WT_ = 6. ^a,b^Columns with different letters differ significantly in pairwise comparisons. (*P* < 0.05). **(b)** Representative original recordings of [Mg^2+^]_i_ changes of Mg^2+^ -preloaded cells measured in completely Mg^2+^-free external buffer solution.

Member A1 of solute carrier family 41 has been extensively characterized as the major Mg^2+^-extrusion system in the plasma membrane^6^,^5^,^21^. The amino acid sequences of A1 and A3 display 56.3% sequence identity and 72.7% sequence similarity (calculated with EMBOSS 6.3.1:matcher, http://mobyle.pasteur.fr/cgi-bin/portal.py?#forms::matcher), respectively. According to the prediction program PSORTII (http://psort.hgc.jp/), the probability for the plasma membrane localisation of A3 is 78.3%, and only 21.7% for its localisation in the endoplasmic reticulum. Based on these data, a functional similarity to the well-characterized plasma membrane Na^+^/Mg^2+^ exchanger A1 was assumed. To examine the anticipated function of A3, we investigated the effect of A3 overexpression on Mg^2+^ fluxes in intact cells. By using the Mg^2+^-sensitive fluorescent dye mag-fura 2, we first examined the ability of SLC41A3 to mediate Mg^2+^ extrusion (efflux condition). Cells were loaded with mag-fura 2 and subsequently incubated in a buffer solution containing 10 mM MgCl_2_ for 20 min. Thereafter, the Mg^2+^-fluxes were measured over a time period of 1,200 s in nominally Mg^2+^-free buffer solution containing 145 mM NaCl. Surprisingly, induced cells of the stable cell line (A3st +tet) and of the transiently transfected cells (Cl2/A3 +tet) did not exhibit Mg^2+^ extrusion but rather an increase in [Mg^2+^]_i_ of 0.26 ± 0.04 and 0.20 ± 0.05 mM/1,200 s (Fig. 2a,b), respectively. In contrast, uninduced A3st (A3st −tet; 0.11 ± 0.02 mM/1,000 s), transiently transfected uninduced Cl2/A3 cells (Cl2/A3 −tet; 0.10 ± 0.02 mM/1,000 s), and wild-type HEK293 cells (HEK WT; 0.05 ± 0.04 mM/1,000 s) exhibited significantly lower changes of [Mg^2+^]_i_ (*P* < 0.05). These data indicate that the increase in [Mg^2+^]_i_ in induced cells was A3 related. Given that the experiments were carried out in completely Mg^2+^-free buffer solution, the increase in the cytoplasmic free Mg^2+^ concentration in SLC41A3-overexpressing cells could not have been caused by Mg^2+^ influx across the plasma membrane.

**Figure 3:**
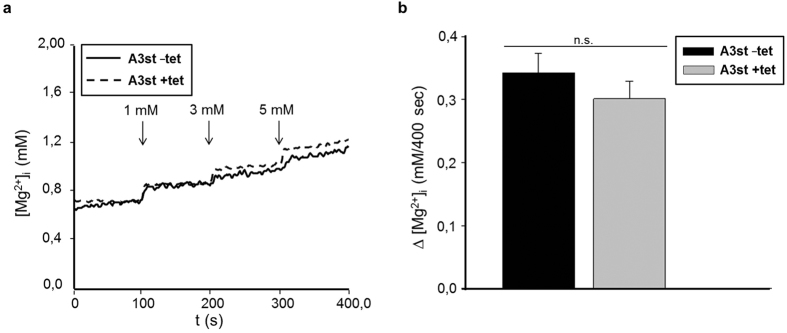
Overexpression of SLC41A3 does not alter the Mg^2+^ uptake capacity across the plasma membrane in HEK293 cells. **(a)** Tetracycline-induced (+tet) or uninduced (−tet) A3st cells were loaded with mag-fura 2, and measurements were carried out in completely Mg^2+^-free buffer solution to which Mg^2+^ was added after 100 s (1 mM), 200 s (3 mM), and 300 s (5 mM final concentration). **(b)** Changes in the intracellular Mg^2+^ concentration (∆ [Mg^2+^]_I_), calculated from averaged concentrations of the first and the last 50 s of the measurement, were 0.34 mM ± 0.03 for uninduced control cells and 0.30 ± 0.03 mM for induced cells. (n.s., not significant).

To substantiate the inability of SLC41A3 to mediate flux of Mg^2+^ across the plasma membrane, Mg^2+^ influx experiments were performed. A3st cells were induced with tetracycline for 24 hours or left untreated and loaded with mag-fura 2. Subsequently, [Mg^2+^]_i_ was measured over a period of 400 s in which Mg^2+^ was added stepwise to final extracellular concentrations of 1, 3 and 5 mM. The shorter duration of 400 s was chosen to minimize the release of Mg^2+^ from intracellular stores. Representative curves for HEK293 cells overexpressing SLC41A3 and for uninduced control cells are shown in Fig. 3a. As expected, in the presence of an inwardly directed Mg^2+^ gradient, −tet and +tet A3st cells exhibited the uptake of the ion into the cell. However, the overexpression of the protein did not result in a significantly different uptake capacity (Fig. 3b), implying that SLC41A3 did not mediate the influx of Mg^2+^ across the plasma membrane under our experimental conditions. Thus, the only logical explanation is that, in response to the overexpression of SLC41A3, Mg^2+^ stored in organelles is released, thereby increasing the cytoplasmic Mg^2+^ concentration.

### SLC41A3 is a mitochondrial protein

**Figure 4:**
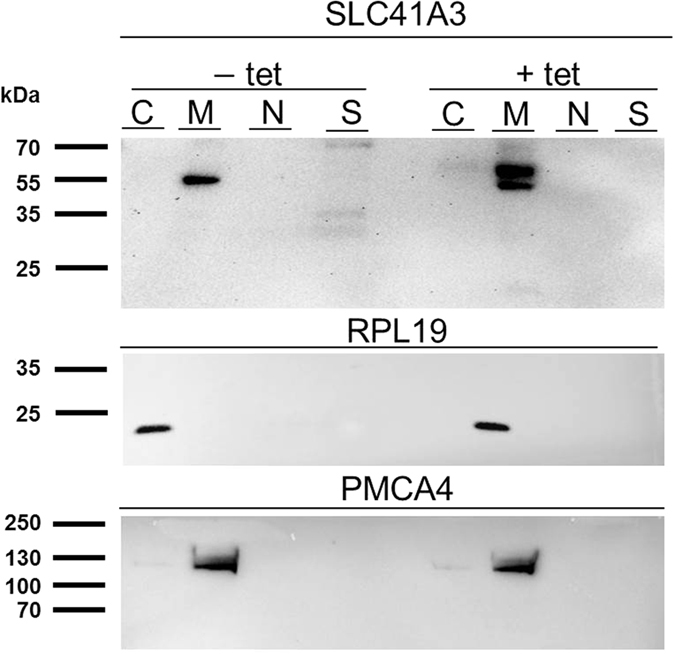
Immunodetection of SLC41A3 after subcellular fractionation. Cytosolic (C), membrane (M), nuclear (N), and cytoskeletal (S) protein-enriched fractions were isolated from −tet and +tet A3st cells and analysed by Western blot. Immunodetection was performed with an antibody directed against native SLC41A3. Immunosignals were detected exclusively in the membrane protein fraction (M) in −tet cells as a single band corresponding to the native protein and, in +tet cells, as two bands corresponding to native and Strep-tagged SLC41A3, respectively. To confirm the specificity of the fractionation, PMCA4 was used as a control for the membrane fraction (M) and RPL19 for the cytoplasmic protein fraction (C).

Next, we performed subcellular fractionation using the Qproteome cell compartment kit (Qiagen) to assess the localisation of SLC41A3 within the cell. In uninduced A3st cells (−tet) a band of ~55 kDa corresponding to A3 was almost exclusively detected in the membrane fraction when using an antibody recognizing native A3 (M; Fig. 4). In induced A3st cells (+tet) detection with the native antibody yielded two signals, one for native A3 and one for the overexpressed Strep-tagged protein. In addition, a weak signal was detected in the fraction containing soluble proteins (C); this signal might have been caused by minor contamination during the sequential isolation of the various fractions. No signal for SLC41A3 was detected in the fractions enriched for nuclear (N) or cytoskeletal proteins (fraction S; Fig. 4). The specificity of the fractionation process was controlled by probing parallel blots with antibodies against the cytosolic ribosomal protein (RP)L19 (fraction S) and the plasma membrane protein PMCA4 (membrane fraction, M). The cytosolic protein RPL19 was detected only in the soluble protein fraction, and the plasma membrane Ca^2+^ ATPase PMCA4 was found, as expected, in the membrane fraction. These data clearly characterize SLC41A3 as being a membrane protein.

**Figure 5:**
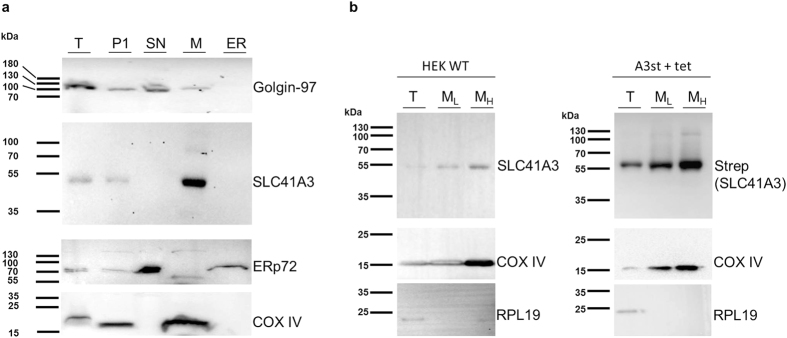
SLC41A3 is primarily localised in mitochondria. **(a**) Western blot analysis of the total protein fraction (T) and the various fractions (pellet 1, P1; supernatant, SN; mitochondrial pellet, M; endoplasmic reticulum, ER) obtained by using an ER isolation kit and differential centrifugation. SLC41A3 was detected with an antibody recognizing the native protein. Golgin-97 served as a Golgi marker, ERp72 as a marker for the ER, and COX IV for mitochondria. **(b)** A mitochondria isolation kit (Sigma-Aldrich) was used to isolate mitochondria-enriched fractions of wild-type HEK293 cells (HEK WT) and of tetracycline-induced A3st (A3st +tet) cells. The total protein fraction (T) was obtained by solubilizing intact cells. The second fraction and third fractions (M_L_ and M_H_) were enriched in mitochondria. The more purified “heavy” fraction M_H_ was obtained by low-speed centrifugation (3,500 *g*), whereas the light M_L_ fraction was isolated by high-speed centrifugation (11,000 *g*). The antibody recognizing native SLC41A3 was used to detect the protein in fractions obtained from HEK WT cells. An anti-Strep antibody was used for the detection of overexpressed SLC41A3 in fractions of A3st +tet cells. Respiratory chain complex IV (COX IV) served as a mitochondrial loading control and the soluble protein RPL19 for the total protein fraction.

As mentioned above, SLC41A3 is predicted to be most likely targeted to the plasma membrane and less probably to the ER. To clarify further the subcellular localisation of SLC41A3 and to exclude potential targeting to the ER, we used an ER isolation kit (Fig. 5a). Various organelle marker proteins were used to control the specificity of the isolation process: Golgin-97 as a Golgi marker, ERp72 for the ER, and COX IV for mitochondria. Most importantly, the experiment was performed with wild-type HEK293 cells, and only endogenous levels of SLC41A3 were detected with an antibody directed against the native protein. As shown in Fig. 5a, various fractions were collected during the differential centrifugation process. SLC41A3 was predominantly detected in fraction M, which mainly contained mitochondrial membranes as verified by the strong signal for COX IV in this fraction. A signal for SLC41A3 was also observed in fraction P1, which contained unbroken cells and plasma membrane. The highly pure ER fraction did not contain SLC41A3. Golgi vesicles were mainly found in fraction SN, which also did not overlap with the signal for native SLC41A3. Taken together, these data clearly argue for the localisation of SLC41A3 in mitochondria and exclude that the protein is targeted to the ER under physiological expression levels.

**Figure 6:**
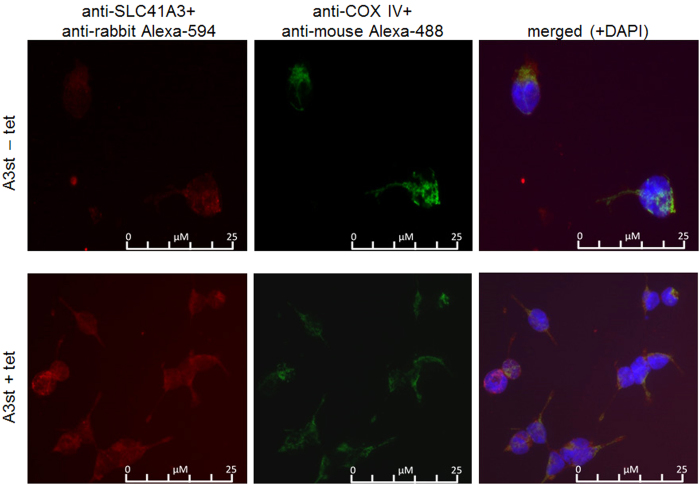
Fluorescence visualization of mitochondrial SLC41A3 localisation. Triple-staining with anti-SLC41A3 antibody (red), anti-COX IV antibody (green) and DAPI (Blue, ony shown in merged) was performed. The merged picture shows that immunosignals for SLC41A3 and COX IV colocalised in uninduced (A3st −tet) and induced (A3st +tet) cells with a stronger intensity upon tetracycline induction.

Next, we sought to confirm the cellular distribution of A3 by fluorescence microscopy. To this end, we performed double-stain immunofluorescence with an antibody directed against the native protein together with an antibody against the mitochondrial marker protein COX IV. Induced and uninduced A3st cells were used to detect potential mistargeting and any aberrant localisation within the cell attributable to the overexpression of the protein (Fig. 6). Strong colocalisation of the two signals was observed in A3st +tet and notably also in A3st −tet cells. These data confirm our previous observations and again suggest that A3 is targeted to mitochondria, both when overexpressed and when under the control of its endogenous promoter. Furthermore, we performed double-stain immunofluorescence in uninduced and induced A3st cells with the anti-Strep antibody in combination with either the mitochondrial marker COX IV, ERp72 for the endoplasmic reticulum or Glogin-97 as Golgi marker. These results are summarized in Supplemental Figs S2–S4. Similar to the results obtained with the native antibody, significant colocalisation was only observed with the mitochondrial protein COX IV (Supplemental Fig. S2).

Finally, we directly isolated mitochondria from HEK WT and A3st +tet cells. After the homogenization step, the obtained suspension was first centrifuged at low speed (3,500 rpm) to obtain a more purified fraction of “heavy” mitochondria. The remaining supernatant was then centrifuged at high speed (10,000 rpm) to yield a fraction enriched in “light” mitochondria. Both fractions were analysed by Western blot, together with the total protein extract. The inner mitochondrial membrane protein COX IV was used as a marker protein to control the specificity of the fractionation process. In the fractions obtained from HEK WT cells, A3 was detected in the two mitochondrial fractions with the antibody recognizing the native protein and was clearly enriched, in particular, in the purer “heavy” mitochondrial fraction compared with the total protein extract (Fig. 5b). In A3st +tet cells, overexpressed A3 was detected with the anti-Strep antibody. The fact that Strep-tagged A3 is enriched particularly in the “heavy” mitochondrial fraction confirmed that also the overexpressed protein is efficiently target to mitochondria.

### Overexpression of SLC41A3 increases the efflux of Mg^2+^ from mitochondria

**Figure 7:**
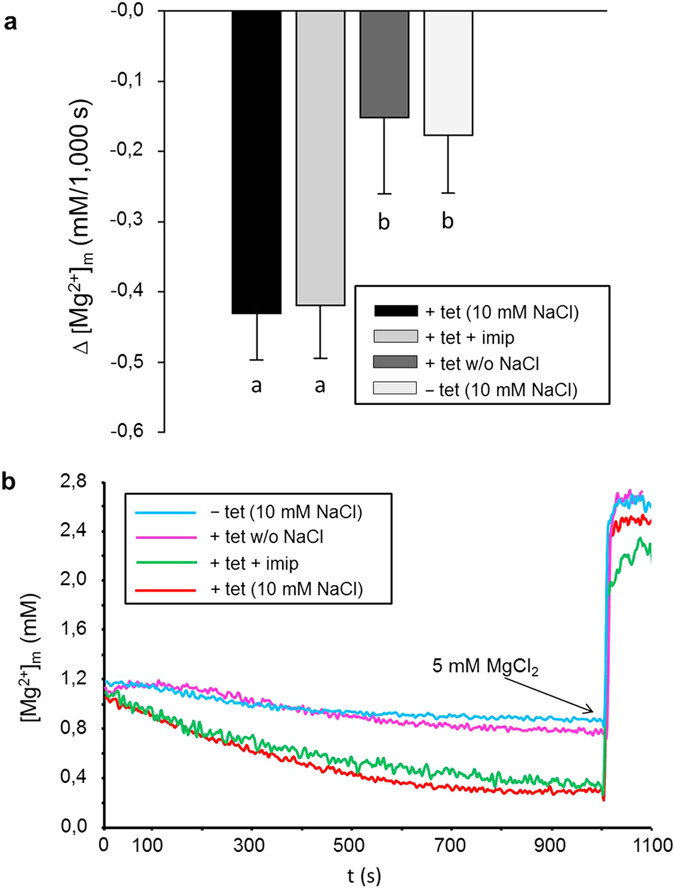
Effect of the overexpression of SLC41A3 on the mitochondrial free magnesium concentration ([Mg^2+^]_m_) in HEK293 cells. **(a**) Mitochondria of tetracycline-induced (+tet) and uninduced (−tet) cells of the SLC41A3 stable cell line A3st were loaded with Mg^2+^, and changes of [Mg^2+^]_m_ during 1,000 s in completely Mg^2+^-free solution were determined. All solutions contained 10 mM NaCl, except solution +tet without NaCl. The imipramine concentration was 250 μM. Values are given as means ± SEM. Number of measurements: N_A3 −tet (10 mM NaCl)_ = 12; N_A3 +tet+imip_ = 6; N_A3 +tet (without NaCl)_ = 5; N_A3 −tet_ = 13; ^a,b^Columns with different letters differ significantly in pairwise comparisons (*P* < 0.01). **(b**) Representative original recordings of [Mg^2+^]_m_ changes of isolated mitochondria in completely Mg^2+^-free medium with or without Na^+^ in the external buffer solution. Before the measurements, mitochondria were loaded with Mg^2+^ by incubating them in Mg^2+^-containing buffer solution (10 mM) for 20 min. Imipramine (imip) was added to the buffer solution directly before measurements were recorded. After 1,000 s, MgCl_2_ was added to give a final concentration of 5 mM, resulting in a steep increase of the mitochondrial [Mg^2+^].

The above presented data clearly argue for the mitochondrial localisation of SLC41A3 and open up the possibility that the protein functions as a Mg^2+^ extrusion system in the inner mitochondrial membrane. To test this hypothesis, we established a “Mg^2+^-loading” protocol for isolated, respiring mitochondria similar to that used successfully to study the function of SLC41A1 in whole cells^6^,^5^. Mitochondrial Mg^2+^ loading takes advantage of the strong inside-negative membrane potential of −150 to −180 mV that is the major driving force for the high capacity Mg^2+^ uptake system, the Mg^2+^-selective channel Mrs2. Incubation of isolated respiring mitochondria in Mg^2+^-containing buffer solutions results in the rapid regulated uptake of the ion. Accordingly, we made use of the observation that isolated mitochondria can be efficiently “loaded” with Mg^2+^ 7 and investigated the effect of overexpression of SLC41A3 on the Mg^2+^ efflux capacity out of the organelle (Fig. 7a,b). Isolated mitochondria were first loaded with the membrane-permeable acetoxymethyl ester (AM) of mag-fura 2 in the presence of 10 mM MgCl_2_, followed by an activation/loading step. During the latter step, the dye was activated by intra-organellar esterases in the presence of MgCl_2_. The intramitochondrial free Mg^2+^-concentration [Mg^2+^]_m_ was then determined over a time period of 1,000 s in nominally Mg^2+^-free buffer solution. After the 1,000 s efflux period, Mg^2+^ was added to give a final concentration of 5 mM in order to demonstrate that the mitochondria maintained their vitality and were able to rapidly increase their free [Mg^2+^]_m_ upon external Mg^2+^ exposure. Figure 7b shows representative curves for mitochondria isolated from overexpressing cells and control cells, whereas Fig. 7a shows summarized data for Δ[Mg^2+^]_m_ over the efflux period. Overexpression of SLC41A3 significantly (*P* < 0.01) increased the efflux capacity by ~55% compared with control cells (−0.43 ± 0.07 mM/1,000 s vs. −0.18 ± 0.08 mM/1,000 s). Moreover, the higher rate of Mg^2+^ efflux was also reflected by the lower free [Mg^2+^]_m_ at the start of each efflux measurement. The starting [Mg^2+^]_m_ (value calculated as the average of the first 50 s of each measurement) of mitochondria isolated from SLC41A3 overexpressing cells was significantly (*P* < 0.05) reduced by 21.2% compared with that of uninduced control cells. This indicates that the mitochondria of SLC41A3 overexpressing cells are loaded less effectively with Mg^2+^ because of a stronger activity of the extrusion system.

### Mg^2+^ efflux from mitochondria overexpressing SLC41A3 is dependent on the presence of Na^+^

**Figure 8:**
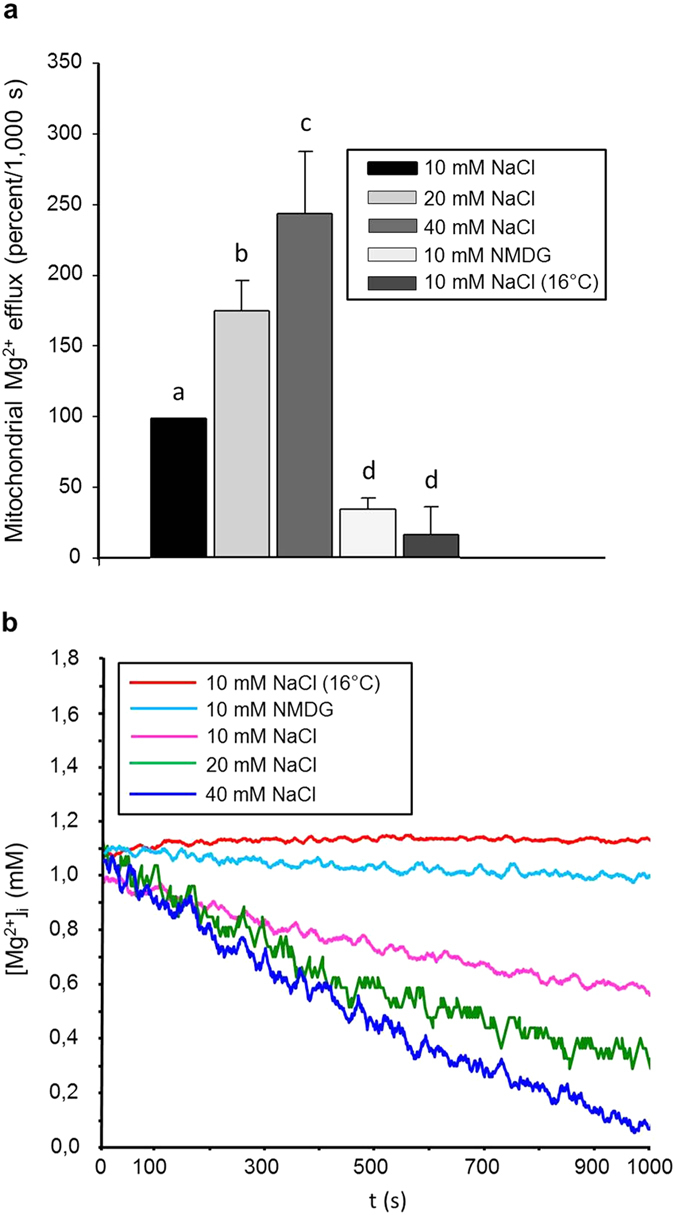
SLC41A3-mediated efflux of Mg^2+^ is dependent on the Na^+^ concentration in the external buffer solution and is temperature-sensitive. **(a)** Mitochondria of tetracycline-induced (+tet) cells of the SLC41A3 stable cell line were loaded with mag-fura2 and Mg^2+^, and the changes of [Mg^2+^]_m_ during 1,000 s in completely Mg^2+^-free solution were determined. The external buffer solution contained various concentrations of NaCl. Values obtained with 10 mM NaCl were set as 100% efflux activity. The efflux capacity increased with increasing NaCl concentrations in the measurement solution. Mitochondria measured at 16 °C exhibited a strongly reduced efflux of Mg^2+^. Similarly, replacement of NaCl with NMDG significantly reduced Mg^2+^ extrusion to approx. 34% of the standard efflux activity. Values are given as means ± SEM. Number of measurements: N_10 mM NaCl_ = 9; N_20 mM NaCl_ = 7; N_40 mM NaCl_ = 7; N_10 mM NMDG_ = 5; N_10 mM NaCl (RT)_ 5. ^a,b,c,d^Columns with different letters differ significantly in pairwise comparisons (*P* < 0.01). **(b)** Representative original recordings of [Mg^2+^]_m_ changes of isolated mitochondria in completely Mg^2+^-free medium with various concentrations of NaCl in the external measurement medium. N-Methyl-D-glucamine (NMDG) was used to replace sodium in the measurement buffer.

Interestingly, the SLC41A3-dependent increase in the efflux capacity was only observed when 10 mM NaCl was present in the external buffer solution. In NaCl-free buffer solution, the efflux capacity of mitochondria isolated from induced cells (−0.15 ± 0.11 mM/1,000 s) was reduced to levels comparable with that of uninduced control mitochondria (Fig. 7). To investigate further the role of Na^+^ in Mg^2+^ extrusion, we performed efflux measurements in the presence of various NaCl concentrations (Fig. 8). Increasing the Na^+^ concentration in the measurement buffer solution to 20 and 40 mM resulted in higher Mg^2+^ extrusion rates compared with measurements performed in the presence of 10 mM NaCl (Fig. 8b). As shown in Fig. 8a, when Mg^2+^ efflux in the presence of 10 mM NaCl was set at 100%, the presence of 20 mM or 40 mM NaCl in the buffer solution increased the efflux capacity to 177 ± 21% and 246 ± 45%, respectively (*P* < 0.01). Next, we investigated the effect of replacing Na^+^ in the measurement solution with N-methyl-D-glucamine (NMDG). The presence of 10 mM NMDG-Cl instead of 10 mM NaCl almost abolished the Mg^2+^ efflux capacity of mitochondria (34.5 ± 7.9%).

### SLC41A3-mediated Mg^2+^ efflux is temperature-sensitive but not affected by imipramine

We furthermore investigated whether imipramine, a known inhibitor of Na^+^/Mg^2+^ exchange mediated via SLC41A1^5^, affected Mg^2+^ extrusion from mitochondria isolated from cells overexpressing SLC41A3. Under our experimental conditions, the application of 250 μM imipramine did not reduce the efflux capacity (Fig. 7). Finally, we tested whether the observed Mg^2+^ efflux was sensitive to changes in incubation temperature. At the lower temperature of 16 °C, Mg^2+^ efflux from mitochondria was almost completely abolished and was similar to that of mitochondria incubated in the absence of Na^+^ (Fig. 8).

### The effect of SLC41A3 overexpression on cellular ATP levels under Mg^2+^ starvation conditions

To get a first insight into the physiological role of SLC41A3 we investigated the effect of A3 overexpression in complete medium and under conditions of reduced Mg^2+^ availability on the total levels of cellular ATP. As cells under culture conditions usually exhibit a high glycolytic activity, thereby masking a potential mitochondrial dysfunction, cells were forced to switch to mitochondrial respiration by replacing glucose/glutamine by galactose/glutamine. A3st cells were seeded and grown for 24 h in DMEM/Gal medium. Thereafter, expression of A3 was induced by addition of tetracycline for another 24 h. The DMEM/Gal medium was then exchanged either for HBSS medium with 0.8 mM MgSO_4_ or for HBSS medium without MgSO_4_. Control cells remained in DMEM/Gal medium. After an incubation period of 8 h a luminescence based assay was performed to determine cellular ATP levels. As shown in Supplemental Fig. S5, relative ATP levels of A3st cells continuously grown in DMEM/Gal or incubated for 8 h in Mg^2+^-containing HBSS medium were unaffected by the expression level of A3. In contrast, cells overexpressing A3 exhibited lower cellular ATP levels than uninduced cells if cultured under Mg limiting conditions. However, due to the high inter-assay variability in luminescence counts the observed reduction in cellular ATP was statistically not significant. Nevertheless, these data imply that overexpression of A3 has a strong tendency to impair mitochondrial ATP production by reducing the Mg^2+^ availability in mitochondria.

## Discussion

Magnesium is vital for normal cellular bioenergetics. In mitochondria, Mg^2+^ not only chelates and stabilizes ATP, but also serves as a cofactor of enzymes involved in cellular respiration and energy production^22^. Moreover, isolated, energized mitochondria are able to accumulate Mg^2+^ up to concentrations that are 3 to 5 times higher than those in cytoplasm^7^. Mg^2+^ has been demonstrated to permeate into the mitochondrial matrix via the high-conductance channel Mrs2, which is powered by a steep negative membrane potential on the inner mitochondrial membrane^7^.

The demonstration that Mrs2 constitutes a major mitochondrial Mg^2+^ influx system adds the desired molecular ratio to the formerly proposed hypothesis that mitochondria represent the major intracellular Mg^2+^ storage compartment^7^,^8^,^10^,^23^. Mitochondria must therefore be a dynamic, tightly regulated, open system able not only to accumulate, but also to release Mg^2+^. Salvi *et al.* demonstrated that besides inducing mitochondrial permeability transition, gliotoxin activates a specific Mg^2+^ efflux system in brain mitochondria^24^. To date, two possible mechanisms of Mg^2+^ release from mitochondria have been suggested: (1) a Mg^2+^ transport (efflux) coupled to ATP translocation via an ATP-MgP_i_ carrier (APC)^25^,^26^,^27^, and (2) a Mg^2+^ efflux system powered by H^+^ motive force^23^,^28^,^29^.

The H^+^ gradient perpetually building-up on the inner mitochondrial membrane represents the major motive force powering the transport of various solutes across the inner mitochondrial membrane. The Na^+^ contribution to the generation of the membrane potential on the inner mitochondrial membrane is thought to be only secondary. However, the role of Na^+^ in the transport physiology of mitochondria is indisputable. Na^+^ homeostasis in mitochondria is governed by transport mechanisms such as the Na^+^/H^+^ exchanger (mNHE, Na^+^ efflux mechanism^30^), Na^+^-HCO_3_^−^ symporter (mNHCO_3_ (SLC4A7), Na^+^ influx mechanism^31^), and the Na^+^/Ca^2+^ exchanger (mNCE, Na^+^ influx mechanism^32^). The paucity of data on the matrix Na^+^ homeostasis in respiring mitochondria leaves the field largely unexplored. Jung *et al.* have reported that, in isolated respiring mitochondria, the [Na^+^]_m_ is approximately 1/8 of that in the cytosol^30^. However, the gradient might be less pronounced for mitochondria *in situ*^33^. Nevertheless, studies of permeabilized cardiac myocytes have confirmed that the matrix [Na^+^]_m_ is lower than cytosolic [Na^+^]_i_ in energized mitochondria^30^,^34^.

In metabolically inhibited, non-permeabilized MDCK cells, [Na^+^]_m_ reaches striking 113 +/−7 mM, which is approximately double the concentration of Na^+^ in the cytoplasm of the same cells^35^. The accumulated Na^+^ is then used to “fuel” the influx of Ca^2+^ via mNCE into the mitochondria^35^. This study has shown that mitochondria possess a large potential to accumulate Na^+^ that can be used to support the transport of other solutes via Na^+^-dependent transport mechanisms under various physiological and pathophysiological situations.

Our data unequivocally demonstrate that SLC41A3 is a protein integral to the inner mitochondrial membrane and that it functions as an Mg^2+^ efflux system coupled with the influx of Na^+^. Based on its similarity with member A1 of the same protein family, namely a Na^+^/Mg^2+^ exchanger integral to the cytoplasmic membrane, we can assume that the coupling between the Mg^2+^ efflux conducted via SLC41A3 and the Na^+^ influx is direct; however, further thorough experiments must be carried out to support this notion. Moreover, we observed a strong temperature dependence of the Mg^2+^ extrusion process mediated by SLC41A3. Reducing the incubation temperature to 16 °C lowered the efflux capacity by ~85% compared with measurements performed at 37 °C. A similar reduction was also observed for the plasma membrane Na^+^/Mg^2+^ exchanger SLC41A1^6^. In contrast, Mg^2+^ influx mediated by the high-conductance channel Mrs2 is entirely insensitive to a reduction of the temperature^7^. This is a further indication that the transport conducted by SLC41A3 is a carrier/exchange mechanism. Interestingly, application of the Na^+^/Mg^2+^ transport inhibitor imipramine had no effect on the SLC41A3-based Mg^2+^ efflux from mitochondria. This is surprising, since imipramine is a potent inhibitor of the plasma membrane localised Na^+^/Mg^2+^ exchanger SLC41A1^5^. However, it can be explained either by stereochemical differences between the Na^+^-binding sites in SLC4A1 and in SLC41A3, or by indirect coupling of SLC41A3-mediated Mg^2+^ efflux to Na^+^ counter-transport. This issue will need further examination.

Many degenerative diseases are hallmarked by a deranged Mg homeostasis at both the cellular and organism levels^36^. In particular, diseases that belong to hereditary or age-related mitopathies have been demonstrated to be characterized by aberrant mitochondrial homeostasis and the consequent loss of control over cellular energy turnover (e.g., Alzheimer’s and Parkinson´s diseases, *diabetes mellitus* type 2, schizophrenia). Furthermore, these diseases are often associated with Mg deficiency^36^,^37^,^38^,^39^,^40^,^41^,^42^. To date, the mitochondrial Mg^2+^ channel Mrs2 is the only transport mechanism that might link Mg deficiency with disturbed mitochondrial homeostasis^43^. Indeed, Kuramoto *et al.* have found that the abrogation of Mrs2 function in the CNS of rats causes massive demyelination and have concluded that normal mitochondrial Mg^2+^ homeostasis is essential for the maintenance of myelin and, thus, CNS functions^44^. Mastrototaro *et al.* have recently suggested a role of the insulin signalling cascade in the regulation of cellular magnesium homeostasis via the Na^+^/Mg^2+^ exchanger SLC41A1 and also via an early onset of Mg^2+^ efflux from intracellular stores, such as mitochondria, the Golgi apparatus, and the endoplasmic reticulum^45^. However, no molecular mechanism could be proposed to explain the efflux of Mg^2+^ from mitochondria. The discovery of SLC41A3 as a mitochondrial Na^+^-dependent Mg^2+^-efflux system now offers the possibility to examine further the effect of insulin on the deposition and reposition of Mg^2+^ under normal and also pathological (diabetic) conditions. Further research on SLC41A3 will lead to the better understanding of the orchestration between extramitochondrial and intramitochondrial Mg homeostasis and their interrelationship with the energy metabolism of the cell. The observation that under Mg^2+^ starvation conditions ATP levels are reduced in SLC41A3 overexpressing cells, suggests an important role of SLC41A3 in mitochondrial energy metabolism. We hypothesise that the joint activity of the Mg^2+^ influx system Mrs2 and the Mg^2+^ efflux system SLC41A3 plays a central role for Mg^2+^ homeostasis in mitochondria. This might be of particular importance for improving the therapeutic strategies and management of age-related mitopathies with simple measures such as Mg supplementation.

## Materials and Methods

### Cell line generation, growth media, and cell culture

To study the localisation and function of SLC41A3, a tetracyclin-(tet)-inducible stably transfected cell line (A3st) was constructed in cooperation with DualSystems Biotech (Schlieren, Switzerland). Briefly, full-length human *SLC41A3* cDNA was cloned into the p*NTGSH* expression vector with an N-terminal HA-Strep tag. The p*NTGSH*-*HA-Strep-SLC41A3* was electroporated into the Flp-In™ T-REx™ HEK293 cell line (Life Technologies, Darmstadt, Germany) and recombined into a defined genomic integration locus that was inserted into the host cell line. Cells were placed under hygromycin B (Hyg) and blasticidin S (Bla) selection in order to select for cells containing the integrated expression construct. Stable resistant clones were harvested and seeded in fresh medium containing Hyg and Bla and finally screened for tet-inducible expression of the HA-Strep-tagged SLC41A3.

The tet-inducible HEK293 cells with stably integrated SLC41A3 were grown at 37 °C under a 5% CO_2_ atmosphere in Dulbecco’s modified Eagle’s medium (DMEM) supplemented with 10% FBS, 1% penicillin/streptomycin (Pen/Strep), 15 μg/mL Bla, and 100 μg/mL Hyg.

To confirm the results obtained in the genetically modified SLC41A3-HEK293 cells, a cell line transiently expressing SLC41A3 was constructed by using the pcDNA5/TO vector system (Life Technologies, Darmstadt, Germany), which allowed tet-inducible expression.

First, HEK293 cells were stably transfected with the plasmid pcDNA6/TR in order to generate a host cell line constitutively expressing the tet-repressor protein. This plasmid also included the Bla resistance gene under the control of the SV40 promoter; expression of this gene allowed the selection of cells stably transfected with the plasmids. We first determined the minimum Bla concentration necessary to kill wild-type HEK 293 cells within 20 days. The selective medium was replenished every 3 days for a time period of 20 days. The appropriate Bla concentration for our HEK 293 cell line was determined to be 15 μg/mL. In the next step, HEK 293 cells were transfected with pcDNA6/TR by using the transfection reagent polyethylenimine (PEI). At 48 hours after the transfection, the cells were serially diluted to obtain single clones able to grow in medium containing Bla (15 μg/mL). Colonies formed under Bla selection integrated the vector stably into their genome and expressed the tet-repressor gene. Several cell foci were picked, further expanded, and tested for tet-inducible gene expression by transiently transfecting them with pcDNA5/TO-SLC41A3. The clone, termed “Clone 2 (Cl2/A3)”, showed the well-regulated expression of SLC41A3 (low background expression in the absence of tet and significant induction of expression upon tet addition).

Cl2/A3 cells were grown at 37 °C and under a 5% CO_2_ atmosphere in DMEM supplemented with 10% FBS, 1% Pen/Strep, and 15 μg/mL Bla.

### Cloning of SLC41A3 into pcDNA™5/TO

The human gene SLC41A3 was synthetized with an N-terminal HA-Strep tag and cloned via 5′ KpnI and 3′ NotI into the expression vector pcDNA™5/TO under the control of a tet-inducible promoter.

### Protein expression and Western blot

SLC41A3 protein expression was induced by the addition of 1 μg/mL tet for 24 hours. After induction, cells were harvested, washed twice in ice-cold phosphate-buffered saline (PBS) and resuspended in lysis buffer (50 mM Tris HCl pH 8.0, 150 mM sodium chloride, 1.2% Triton X-100, 0.1% SDS, 1 mM EDTA, and protease inhibitor cocktail (cOmplete mini, EDTA-free, Roche Diagnostics)). Lysis was performed for 20 min at 4 °C with gentle agitation followed by a clarifying spin (20 min, 14,000 rpm, 4 °C). The supernatant was then resolved on a 10% polyacrylamide-gel (SDS-PAGE). Following electrophoresis, semi-dry blotting to a polyvinylidene difluoride (PVDF) membrane was performed. A primary mouse antibody directed against the Strep-tag (1:2,500, Qiagen, Hilden, Germany) or a rabbit antibody against the N-terminus of native SLC41A3 (Santa Cruz Biotechnology, Heidelberg, Germany) in combination with the respective horseradish peroxidase (HRP)-conjugated secondary antibodies (anti-mouse, 1:1,000; anti-rabbit, 1:2,000; both from Cell Signaling Technology, Frankfurt, Germany) were used to detect SLC41A3. Proteins were visualized by use of the SuperSignal^TM^ West Dura system (Pierce, Dreieich, Germany).

### Blocking peptide competition assay with the antibody recognizing native SLC41A3

The SLC41A3-specific rabbit antibody was purchased from Santa Cruz Biotechnology (Heidelberg, Germany) together with the blocking peptide. To test the specificity of the antibody a competition assay was performed. Total protein samples (5 and 15 μg) of HEK293 wild type cells and of induced A3st cells were separated in triplicate on two 8.5% SDS-PAA gels and blotted to a PVDF membrane. Each membrane was cut into three strips. The first strip was incubated with the SLC41A3-specific antibody at a concentration of 0.125 μg/μl in 2.5% milk/TBS-T. The second strip was incubated in a mixture of anti-SLC41A3 antibody (0.125 μg/ml) and blocking peptide (0.25 μg/μl). The third membrane strip was incubated with a mixture of anti-SLC41A3 antibody and blocking peptide with the concentrations mentioned above together with anti-Strep antibody (0.8 μg/μl). After over-night incubation of the membranes with the primary antibody, one membrane was incubated with the anti-rabbit secondary antibody (dilution: 1:2,000, suitable for the anti-SLC41A3 antibody); the second membrane was incubated with the secondary anti-mouse antibody (dilution 1:1,000, suitable for the primary anti-Strep antibody). Proteins were visualized by use of the Clarity™ Western ECL Blotting Substrate (Bio-Rad, Munich, Germany).

### Gross cell-compartment specific localisation of SLC41A3

The Qproteome Cell Compartment Kit (Qiagen) was used according to the manufacturer’s instructions to isolate sequentially the proteins associated with the cytosol, nucleus, cellular membranes, or cytoskeleton. The various fractions were analysed by Western blotting. For detection of SLC41A3, the above-described antibody recognizing the native protein was used. Specificity of the isolation was controlled with a mouse antibody against the cytosolic marker protein RPL19 (cytosolic ribosomal protein (RP)L19, Abnova, Heidelberg, Germany) and a mouse antibody directed against the plasma membrane Ca^2+^-ATPase (PMCA4, Sigma-Aldrich, Munich, Germany). Secondary antibodies were the same as those mentioned above.

### Isolation of various cellular membranes/organelles from HEK293 cells

The Endoplasmic Reticulum Isolation Kit (Sigma-Aldrich, Munich, Germany) was used for the isolation of intracellular organelles by differential centrifugation. The experiments were performed according to the manufacturer’s protocol. In brief, 3 × 10^8^ wild-type HEK293 cells were collected and washed with PBS. Cells were first resuspended in hypotonic extraction buffer, incubated for 20 min at 4 °C, and centrifuged again. The pellet was resuspended in isotonic extraction buffer, and cells were homogenized by using a Dounce homogenizer. An aliquot of the homogenate was saved for Western blot analysis (total protein fraction in Fig. 5a). The homogenate was centrifuged at 1,000 *g* and 4 °C for 10 min. The post-nuclear supernatant was transferred to a new tube. An aliquot was stored for later analysis (fraction SN in Fig. 5a). The remaining supernatant was centrifuged at 12,000 *g* and 4 °C for 15 min. The supernatant represented the post-mitochondrial fraction, whereas the pellet contained the mitochondrial membranes. The pellet was therefore stored for protein extraction and further analysis (fraction M in Fig. 5a). From the post-mitochondrial supernatant, ER-enriched microsomes were prepared by CaCl_2_ precipitation according to the protocol (fraction ER in Fig. 5a). For Western blot analysis, supernatant fractions were mixed with 4× SDS-sample buffer, and proteins from the pellet fractions were extracted with standard RIPA buffer. Proteins were resolved on 10% polyacrylamide-gels (SDS-PAGE) and blotted to polyvinylidene difluoride (PVDF) membranes. The above-described rabbit antibody was used for detecting native SLC41A3. Antibodies for controlling the specificity of the fractionation were: Golgin-97 as a Golgi network marker, protein disulphide isomerase family A member 4 (ERp72) as an ER marker, and cytochrome c oxidase (COX IV) for mitochondria (all from Cell Signaling Technology, Frankfurt, Germany). Secondary antibodies were used as mentioned above.

### Isolation of mitochondria for protein analysis

For the small-scale isolation of an enriched mitochondrial fraction, the Mitoiso2 kit for cultured cells (Sigma-Aldrich, Taufkirchen, Germany) was used according to the manufacturer’s protocol. All experiments were performed according to the “homogenization” method. The first “heavy” mitochondrial fraction was obtained by centrifugation at 3,500 *g*, and the remaining supernatant was then centrifuged at 11,000 *g,* yielding the “light” mitochondrial fraction. The obtained fractions were analysed by Western blotting either with the antibody recognizing native SLC41A3 or with the antibody directed against the Strep tag as described previously. RPL19 (mouse anti-RPL19, Abnova, Heidelberg, Germany) was used as a cytosolic marker protein and cytochrome c oxidase (rabbit anti-CoxIV, Cell Signaling Technology, Frankfurt, Germany) as a mitochondrial marker. Secondary antibodies were the same as those mentioned above.

### Fluorescence microscopy

Cells were grown on glass coverslips in 12-well plates. At 80% confluence, protein expression was induced by the addition of tetracycline. At 24 hours after induction, cells were treated 15 minutes with 4% paraformaldehyde and then washed 3 times in PBS. Blocking was performed with 10% normal goat serum (NGS) in PBS for 1 hour. The cells were then incubated for 1 hour with the primary antibody against native SLC41A3 diluted 1:500 in 1% NGS, washed three times in PBS and finally incubated 1 hour with a goat anti-rabbit secondary antibody labelled with Alexa red-fluorescent dye (excitation at 561 or 594 nm) at a dilution of 1:500 in 1% NGS. Subsequently, the cells were incubated with a primary antibody against the mitochondrial protein COX IV diluted 1:200, washed three times in PBS and then incubated with a goat anti-mouse secondary antibody labelled with Alexa green-fluorescent dye (excitation at 488 nm) at a dilution of 1:500 in 1% NGS.

Double-stain immunofluorescence was performed also with the two primary antibodies anti-Strep (1:500) and anti-COX IV (1:1000) with the respective secondary antibodies conjugated to Alexa green-fluorescent and Alexa red-fluorescent dyes.

Another aliquot of cells were immunostained with a primary antibody against Strep-tagged SLC41A3 (1:500) and a secondary goat anti-mouse antibody (1:500), followed by an incubation with the primary antibody specific for the Golgi marker protein Golgin-97 (1:100) or the ER marker protein ERp72 (1:100) followed by a secondary goat anti-rabbit antibody (1:500).

Finally the cells were washed in PBS. The coverslips were mounted with the mounting medium Fluoroshield with DAPI (Sigma-Aldrich, Munich, Germany) to visualize cell nuclei and inverted onto glass slides suitable for microscopy. Digital images were acquired with an automated inverted microscope (Leica DMI 6000 B) and analysed with the microscope imaging software Las AF (Leica).

### Quantification of intracellular Mg^2+^

In order to characterize the Mg^2+^ transport activity of SLC41A3, the Mg^2+^-sensitive fluorescent dye mag-fura 2 was used under influx or efflux conditions. [Mg^2+^]_i_ was determined by measuring the fluorescence of the mag-fura-2-loaded cells in an LS55 spectrofluorometer (PerkinElmer) by using the fast filter application with alternating excitation at 340 nm and 380 nm and emission at 515 nm. SLC41A3-HEK cells (stably transfected) were grown to 80% confluence; protein expression was induced by the addition of tetracycline (tet, 1 μg/mL) for 24 hours. Subsequently, cells were gently scraped, washed in Ca^2+^- and Mg^2+^-free HBSS (137 mM NaCl; 5.36 mM KCl; 0.34 mM Na_2_HPO_4_; 0.44 mM KH_2_HPO_4_; 5.55 mM glucose; 4.17 mM NaHCO_3_, 20 mM Hepes) and then loaded for 20 min with mag-fura 2 AM (7.5 μM) on a shaking plate at 37 °C. Following mag-fura 2 loading, the cells were incubated for another 20 min at 37 °C in HBSS to allow the complete de-esterification of the fluorescent probe, washed twice with HBSS to remove extracellular mag-fura 2, and resuspended in completely Mg^2+^- and Ca^2+^-free HBSS. Measurements of Mg^2+^ influx were performed at 37 °C in 3-ml cuvettes containing 2 ml cell suspension that was constantly stirred. Extracellular Mg^2+^ was added stepwise at increasing concentrations, ranging from 1 to 5 mM, during the measurement. For the Mg^2+^ efflux experiments, the cells were incubated with 10 mM MgCl_2_ at 37 °C for 20 min after mag-fura 2 loading, washed twice in Mg^2+^- and Ca^2+^-free HBSS, and finally measured in Mg^2+^- and Ca^2+^-free HBSS at 37 °C in 3-ml cuvettes.

### Large-scale isolation of mitochondria for mag-fura 2 measurements

Cells were harvested in ice-cold PBS, centrifuged, and washed in ice-cold isolation buffer (IB; 210 mM Mannitol, 70 mM sucrose, 5 mM Hepes-KOH pH 7.2, and 0.5% BSA). The cell pellet was resuspended (4 mL IB/g cells), and digitonin (10 mg/mL in DMSO) was added stepwise to permeabilise the cells. The permeabilisation efficiency was controlled by trypan blue staining. After sufficient permeabilisation was reached, 5 ml IB was added, and the suspension was centrifuged (3,000 *g*, 5 min, 4 °C). The pellet was resuspended in IB, and the suspension was homogenized by using a Dounce homogenizer. IB without BSA (3 times the volume of the homogenate) was added, and unbroken cells and cell debris were pelleted by centrifugation (1,200 *g*, 3 min, 4 °C). This clarifying step was repeated once before the suspension was finally centrifuged (10,000 *g*, 20 min, 4 °C) to pellet the mitochondria. The mitochondrial pellet was resuspended in 1 mL IB without BSA supplemented with 0.5 mM ATP, 0.2% succinate, and 0.01% pyruvate.

### Quantification of free magnesium in mitochondria

Mitochondria were loaded with mag-fura 2 AM (7.5 μM) for 20 min on a shaking plate at 37 °C in IB without BSA+S (S: supplemented with 0.5 mM ATP, 0.2% succinate, and 0.01% pyruvate). Following mag-fura 2 loading, mitochondria were incubated in IB without BSA+S with 10 mM MgCl_2_ at 37 °C for 20 min (mag-fura 2 AM activation) and then washed twice in Mg^2+^ and Ca^2+^-free IB without BSA+S. Finally, [Mg^2+^]_i_ was determined in IB without BSA+S supplemented with 10 mM NaCl in an LS55 spectrofluorometer as described above. The osmolarity of the HBSS measurement buffer solutions with various NaCl concentrations or NMDG-Cl was controlled and maintained between 290 and 300 mosmol/L. Imipramine was dissolved in water and added to give a final concentration of 250 μM during the dye activation step and the measurements. Curves of representative recordings were smoothened with a “moving average” algorithm (*F*_s_ = 99; FL WinLab version 4.00.03).

### Determination of relative cellular ATP levels

A luminescence ATP detection assay kit (Abcam, Cambridge, UK) was used to determine relative cellular ATP levels in uninduced and induced A3st cells under various growth conditions. Cells were seeded in duplicate in 24-well plates at a starting density of 8000 cells/well in glucose/glutamine-free DMEM medium (Biochrom, Berlin, Germany). The medium was supplemented with 5 mM D-galactose, 6 mM L-glutamine, 1 mM sodium pyruvate, 10% dialyzed FBS, 15 μg/mL Bla, and 100 μg/mL Hyg. Cells were cultured for 24 hours, and then expression of A3 was induced by addition of tetracycline. Control cells were left untreated. After 24 hours the medium was exchanged either against Mg^2+^-free HBSS medium (1x HBSS salts, 5 mM D-galactose, 6 mM L-glutamine, 1 mM sodium pyruvate, 10% dialyzed FBS, 15 μg/mL Bla, and 100 μg/mL Hyg) or HBSS medium (same composition as above) but supplemented with 0.8 mM MgSO_4_. Control cells remained in the aforementioned DMEM medium. After another 6 hours of incubation the ATP assay was performed as follows. Two-hundred μl of the supplied detergent solution was directly added to the medium and the plate was incubated for 5 min in an orbital shaker. Then 200 μl of substrate solution was added to each well and the plate was incubated for another 5 min under constant shaking. The suspension of each well was then transferred to four wells of a 96 well plate (200 μl each) and after 10 min of dark adaptation luminescence was measured with an EnSpire multimode plate reader (PerkinElmer). Results were blank corrected against wells without cells but containing medium, detergent and substrate solution. The experiment was performed two times.

### Statistical analyses

(1) A two-tailed Student’s t-test was used to compare the differences between two means (i.e., influx in induced and uninduced cells, Fig. 3b). (2) A post hoc Holm-Sidak one-factor ANOVA (all pairwise multiple comparison) was used when three or more groups were compared (i.e., efflux experiments in induced and uninduced stable and transient cell lines in Fig. 2 or mitochondrial efflux, Fig. 7a). (3) A post hoc Dunn’s one-factor ANOVA (multiple comparisons versus control group) was used when three or more groups were compared with a control (i.e., mitochondria efflux under various NaCl concentrations or temperature conditions, Fig. 8). (4) A post hoc Holm-Sidak two-factor ANOVA (all pairwise multiple comparison) was used to compare 2 factorial data sets (i.e., tet x different media for the determination of cellular ATP levels, Supplemental Fig. S5).

A Shapiro-Wilk normality test was used for (1), (2) and (3). Data are presented as means ± SE. Differences of *P* < 0.05 were considered significant. Statistical analyses were executed by using SigmaPlot 11.0 (Systat Software, Inc.).

## Additional Information

**How to cite this article**: Mastrototaro, L. *et al.* Solute carrier 41A3 encodes for a mitochondrial Mg^2+^ efflux system. *Sci. Rep.* **6**, 27999; doi: 10.1038/srep27999 (2016).

## Supplementary Material

Supplementary Information
