# Supplementary material for: Solute carrier 41A3 encodes for a mitochondrial Mg2+ efflux system
Source: Sci Rep. 2016 Jun 15;6:27999. doi: 10.1038/srep27999 (PMC4908428; doi:10.1038/srep27999)
Supplement: Supplementary Information [file srep27999-s1.pdf]

Solute carrier 41A3 encodes for a mitochondrial Mg^{2+} efflux system

Lucia Mastrototaro, Alina Smorodchenko, Jörg R. Aschenbach, Martin Kolisek, Gerhard Sponder

Supplementary Information

Supplementary Figures

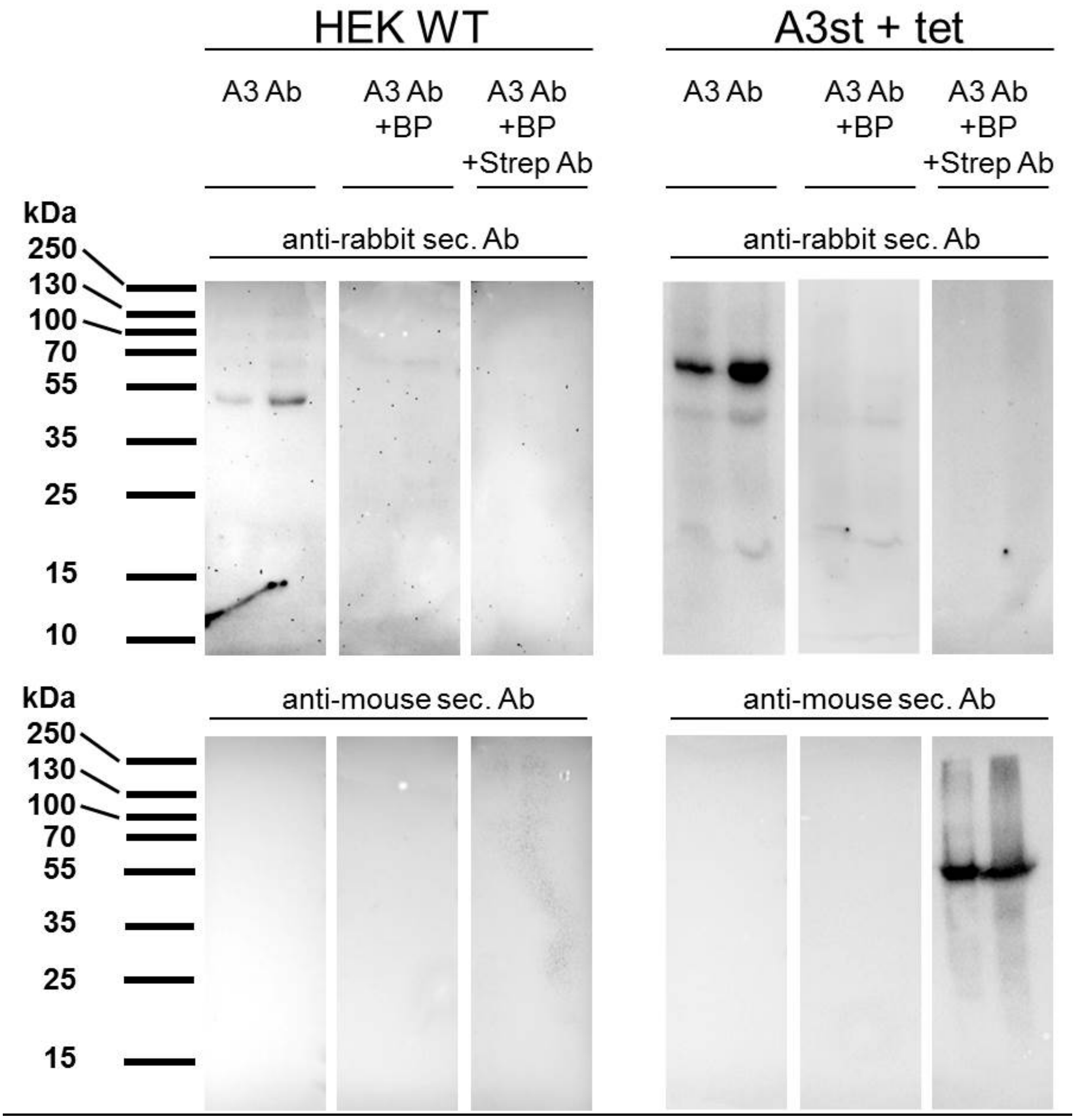

Supplementary Figure S1–Blocking peptide competition assay to verify the specificity of the antibody recognizing native SLC41A3.

Total protein extracts (left lane: 5 µg, right lane: 15 µg) of wild-type HEK293 cells (HEK WT) or tet-induced A3st cells (A3st + tet) were separated in triplicates on two 8.5% SDS-PAA gels and blotted on PVDF membranes. Membrane strips were either incubated with the anti-rabbit SLC41A3 antibody (A3 Ab), with a mixture of A3 Ab and a SLC41A3-specific blocking peptide (BP) or with a mixture of A3 Ab, the blocking peptide, and mouse anti-Strep Ab. After the primary incubation step, the first membrane was incubated with a secondary anti-rabbit antibody, the second membrane with an anti-mouse secondary antibody. Finally, proteins were visualized by chemiluminescence. In HEK WT cells (left panel) the A3 Ab efficiently recognized native SLC41A3 only in the absence of the blocking peptide. In A3st + tet cells (right panel) the A3 antibody recognized native SLC41A3 and the overexpressed Strep-tagged protein, no signal was detected in the presence of the blocking peptide. In contrary, the blocking peptide did not inhibit binding of the Strep-antibody to Strep-SLC41A3.

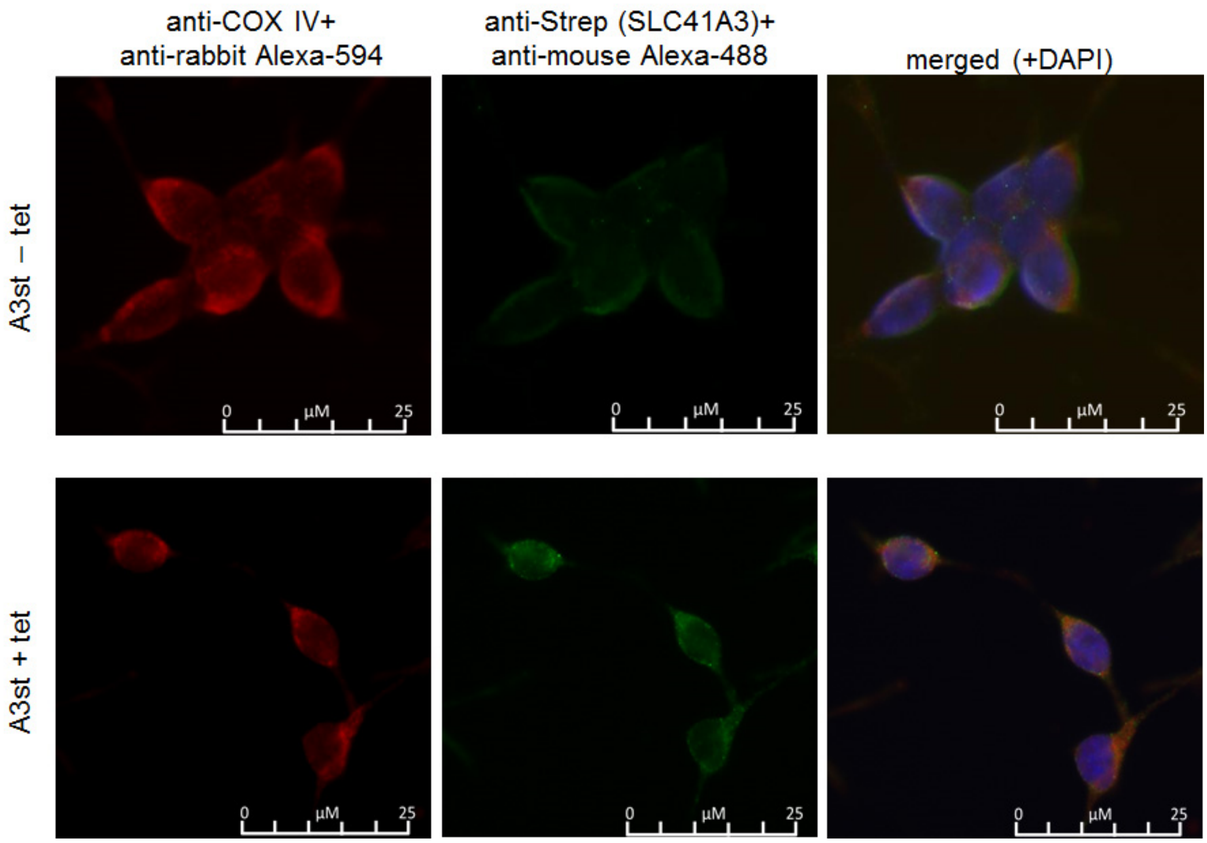

Supplementary Figure S2 – Fluorescence visualization of Strep-SLC41A3 and COX IV.

Triple-staining with anti-SLC41A3 antibody (red), anti-COX IV antibody (green), and DAPI (blue, only shown in merged) was performed in uninduced (A3st -tet) and induced (A3st +tet).

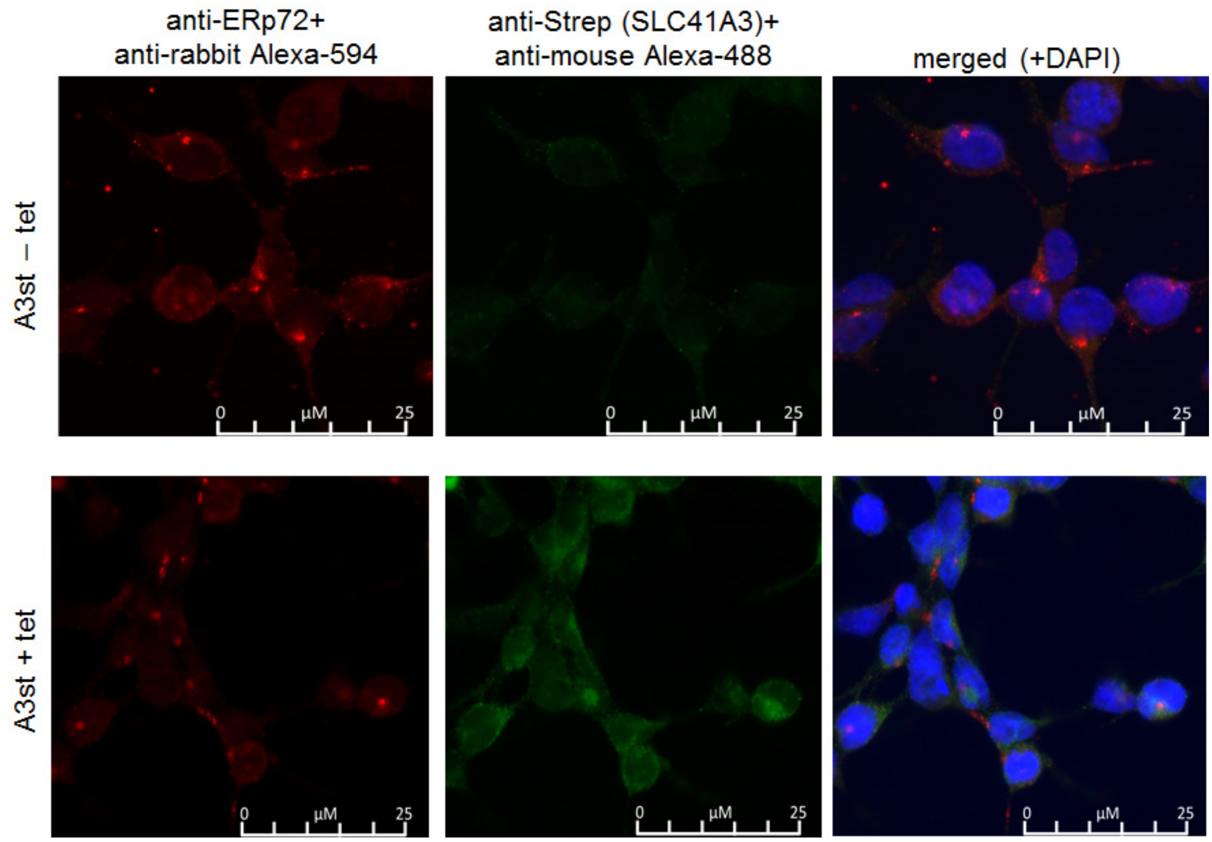

Supplementary Figure S3 – Fluorescence visualization of Strep-SLC41A3 and ERp72.

Triple-staining with anti-SLC41A3 antibody (red), anti-ERp72 antibody (green), and DAPI (blue, only shown in merged) was performed in uninduced (A3st -tet) and induced (A3st +tet).

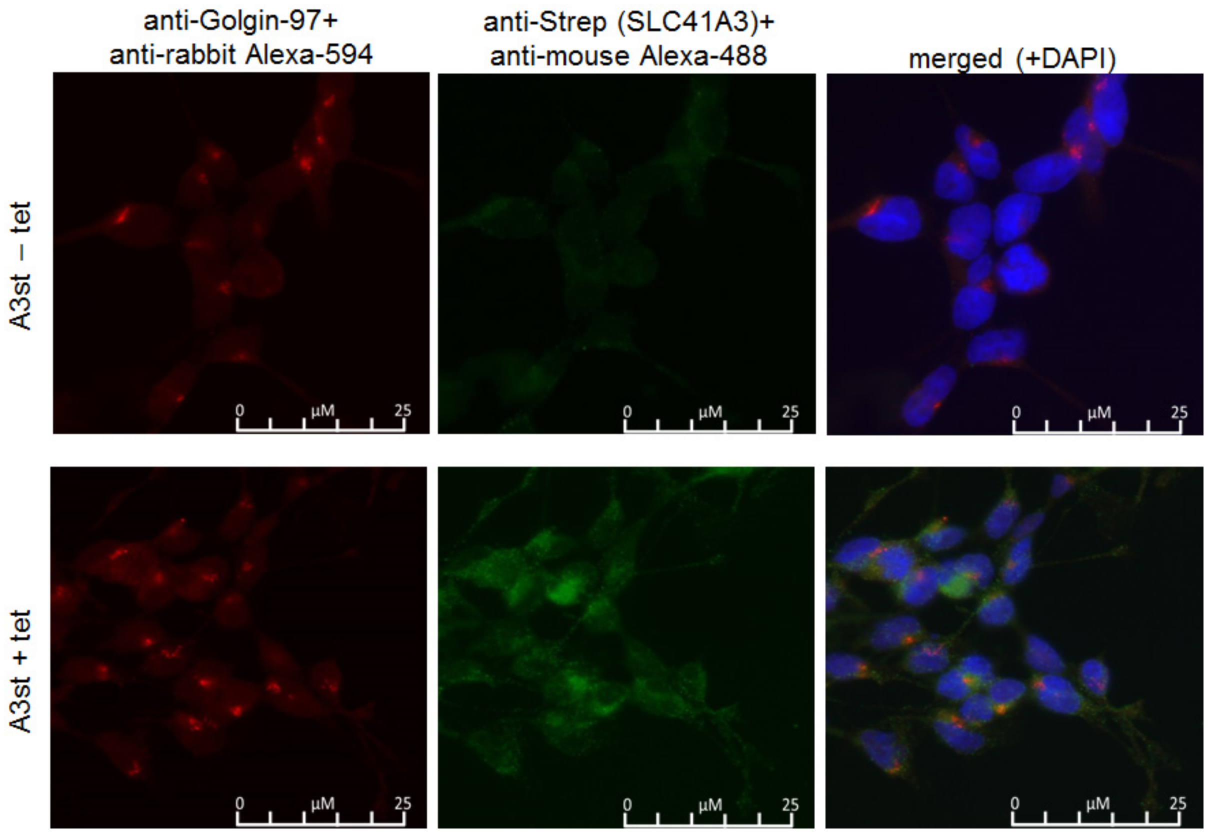

Supplementary Figure S4 – Fluorescence visualization of Strep-SLC41A3 and Golgin-97.

Double-staining with anti-SLC41A3 antibody (red), anti-Golgin-97 antibody (green), and DAPI (blue, only shown in merged) was performed in uninduced (A3st -tet) and induced (A3st +tet).

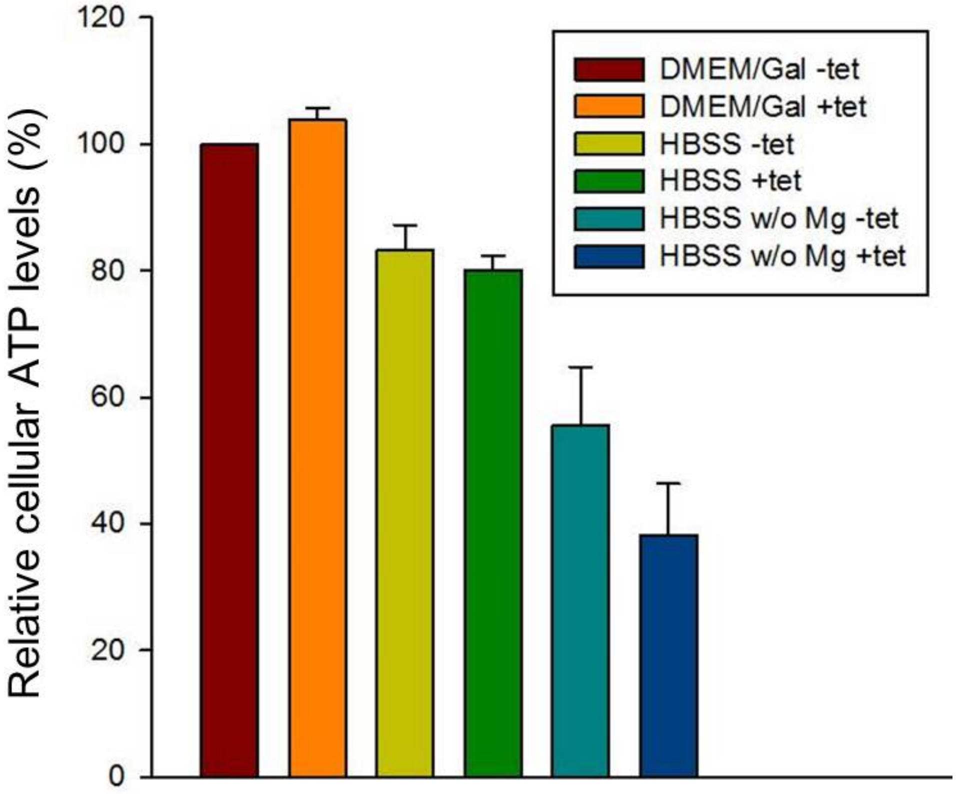

Supplementary Figure S5 – Relative cellular ATP levels in uninduced and induced A3st cells under various culture conditions.

ATP levels of uninduced (-tet) cells continuously grown in DMEM/Gal medium served as a control (100%), all other experimental conditions are given as percentage relative to the control level. Overexpression of A3 (+tet) in DMEM/Gal medium did not reduce ATP levels. In both uninduced and induced cells, HBSS medium containing $MgSO_4$ reduced cellular ATP by approximately 20%. A further reduction of cellular ATP was seen in Mg^{2+}-starvation medium which was more pronounced in A3 overexpressing cells.
